# Shortened oral contrast preparation for improved small bowel distension at MR enterography

**DOI:** 10.1007/s00261-017-1133-4

**Published:** 2017-04-09

**Authors:** M. I. J. Bekendam, C. A. J. Puylaert, S. K. S. S. Phoa, C. Y. Nio, J. Stoker

**Affiliations:** Department of Radiology, Academic Medical Center, Amsterdam, The Netherlands

**Keywords:** MRI, MR enterography, Bowel distension, Small bowel, Oral contrast preparation

## Abstract

**Purpose:**

Adequate small bowel distension in MR enterography is important for the evaluation of disease activity in Crohn’s disease patients. While distension of the distal small bowel can be achieved using standard oral contrast preparation, proximal small bowel distension remains a common impediment. The aim of this study was to compare small bowel distension between a 60-min oral contrast preparation and a 45-min oral contrast preparation.

**Methods:**

Fifty retrospectively included patients with a 60-min oral preparation protocol and 50 prospectively included patients with a 45-min three-portion oral preparation protocol were included in the study. Both groups gradually ingested a total of 1600 mL 2% Mannitol solution during the preparation time. Two observers independently graded distension of the stomach, duodenum, jejunum, ileum, and (neo-) terminal ileum. Total small bowel distension was calculated as the sum of all small bowel segment scores. Individual and averaged observer distension scores were compared between both groups of patients using *χ* ^2^ test for ordinal variables.

**Results:**

Significant differences in distension for one of both observers in favor of the 45-min protocol were found for the stomach (*p* = 0.04), duodenum (*p* = 0.02), jejunum (*p* = 0.02), and total small bowel (*p* = 0.02). When distension scores were averaged between observers, the stomach, jejunum, and total small bowel showed a significant difference in favor of the 45-min protocol (*p* = 0.04, 0.02, and 0.02, respectively).

**Conclusion:**

We advise to use a 45-min three-portion oral preparation protocol for MR enterography for improved overall small bowel distension, proximal small bowel distension, and especially jejunal distension.

Magnetic resonance (MR) imaging is commonly used for establishing small bowel disease activity in Crohn’s disease patients [1]. Assessment of MR disease parameters, such as bowel wall thickening and post-contrast enhancement, depends on adequate small bowel distension obtained by luminal contrast medium [2]. Two techniques exist for administration of luminal contrast medium: MR enterography and MR enteroclysis. Using MR enterography, distension is achieved by oral administration of luminal contrast medium, while in MR enteroclysis distension is achieved by administration of luminal contrast medium through a nasojejunal tube [3, 4]. A previous study found similar distension and diagnostic accuracy for active Crohn’s disease in the terminal ileum when comparing MR enterography and MR enteroclysis with the use of an endoscopic reference standard [5]. MR enteroclysis showed improved proximal small bowel distension, although no difference in diagnostic yield was seen. Only 5 patients were diagnosed to have lesions in a total of 80 proximal small bowel segments. Therefore, the low yield of proximal small bowel disease in this study prohibits any firm conclusions on the effect of proximal small bowel distension on diagnostic accuracy. Benefits of MR enterography are improved comfort and no burden of ionizing radiation as nasojejunal tube placement is not necessary [6, 7]. Consequently, MR enterography is preferred for small bowel evaluation by most institutions [5–8].

In clinical practice, institutions use a variety of preparation protocols for MR enterography as was shown in a recent meta-analysis [9]. Furthermore, the recently published European Society of Gastrointestinal Abdominal Radiology technical guideline recommends an ingestion period for oral contrast medium of 45–60 min prior to the MR examination for adequate small bowel distension [10]. At our institution, until recently a 60-min oral preparation protocol was used, which generally provided adequate distension of the distal small bowel. However, in our experience, distension of the proximal small bowel is often inadequate. In such cases, progression of luminal contrast medium into colonic segments can be found. In addition, colonic distension is not the primary goal of MR enterography and can be seen as an unwanted loss of oral contrast medium for proximal small bowel distension. This loss could indicate an inappropriate timing of contrast administration [11, 12]. Therefore, we hypothesize that a shortened oral contrast administration will improve proximal small bowel distension without reducing distal small bowel distension. The aim of this study was to compare small bowel distension between a 60-min oral contrast preparation and a 45-min oral contrast preparation.

## Materials and methods

### Patients

In December 2015, the oral contrast preparation protocol for MR enterography at our institution was changed from a 60-min preparation to a 45-min preparation. Between December 2015 and March 2016, we included 50 consecutive patients who underwent MR enterography for their clinical work-up as suspected or known Crohn’s disease patients using the 45-min preparation protocol, while we retrospectively included a consecutive group of 50 patients from June 2015 to October 2015, who had used the 60-min preparation. Patient exclusion criteria were the same for both groups: age younger than 18 years, contraindications for MRI (e.g., pacemakers, claustrophobia), more than one examination in the inclusion period (only the first examination was included), previous gastrointestinal surgical procedures with severe impact on grading of small bowel distension (e.g., previous gastrojejunostomy, ileostomy, or short bowel syndrome), non-compliance to the oral preparation protocol and lastly the use of a divergent scan or preparation protocols for research studies (as these differences could impact distension). Informed consent was waived by the hospital’s medical ethics committee as all patients underwent MR enterography as part of their clinical work-up.

### Preparation protocols

*60*-*min preparation* Patients fasted for 4 h prior to the MRI examination and were instructed to ingest 1600 mL of 2% Mannitol (Baxter, Utrecht, The Netherlands) solution starting 60 min before the examination. Furthermore, patients were instructed to gradually ingest the oral contrast solution (e.g., to use the entire length of the preparation time for ingestion). The following data were retrieved: time of registration, time of start scan, sex, age, and relevant surgical and clinical history. Exact drinking times and administered volume were not documented at that time. However, an adequate estimation of drinking time could be made using the difference between the arrival time at the MRI-unit and time at start of the examination.

*45*-*min preparation* Patients fasted for 4 h prior to the MRI examination and were instructed to ingest 1600 mL of 2% Mannitol solution in three portions, starting 45 min before the scan. Furthermore, patients were instructed to gradually ingest the oral contrast solution and to ingest portion three directly before the scan. The following data were documented: relevant surgical and clinical history, drinking time, ingested volume from portion one (700 mL; 0–20 min), portion two (700 mL; 20–40 min), and portion three (200 mL; directly before scan).

### MR imaging protocol

Patients were scanned feet first in supine position on a 1.5 T MR unit (MAGNETOM Avanto, Siemens, Erlangen, Germany) using dual-phased array coils. The imaging protocol consisted of coronal and axial balanced gradient-echo (GE) sequences, followed by an axial fat-saturated, T2-weighted single-shot fast spin echo (SSFSE) and lastly, fat-saturated T1-weighted spoiled gradient echo (SPGE) in the coronal plane (non-enhanced) and in axial and coronal planes 60 s after injection of intravenous gadobutrol (Gadovist 1.0 mmol/L, Bayer Schering Pharma, Berlin, Germany). Specific MR sequence parameters are given in Table 1. To reduce bowel peristalsis, 20 mg of butylscopolamine bromide (Buscopan, Boehringer, Ingelheim, Germany) was administered intravenously before intravenous contrast injection [10]. In case of contraindication to butylscopolamine bromide (e.g., glaucoma or prostatic hypertrophy), 1.0 mg of glucagon (GlucaGen HypoKit 1 mg, Eureco-Pharma BV, Ridderkerk, The Netherlands) was used.

**Table 1:** MR scan protocol at 1.5T

	Balanced GE	Balanced GE	T2-w SSFSE	3D T1-w SPGE*	3D T1-w SPGE**
Plane	Coronal	Axial	Axial	Coronal	Axial
Fat saturation	No	No	Yes	Yes	Yes
TR (ms)	3.75	4.11	1400	4.74	4.74
TE (ms)	1.88	2.06	93	2.38	2.38
Flip angle (°)	70	70	160	10	10
Slice thickness/gap (mm)	5/0	5/0	6/1.2	3/0	3/0
Slices	23	40	60	72	88
Field of view (mm)	500 × 422	350 × 296	380 × 380	500 × 360	450 × 352

ms, milliseconds; °, degree of angle; mm, millimeter; GE, Gradient echo; T1-w T1, weighted MRI sequence; T2-w T2, weighted MRI sequence; SSFSE, single-shot fast spin echo; SPGE, spoiled gradient echo; *, before and after intravenous contrast administration; **, only after intravenous contrast administration

### Image analysis

For MRI evaluation, the following five segments were evaluated: stomach, duodenum, jejunum, ileum, and (neo-) terminal ileum. Each scan was independently assessed by two experienced abdominal radiologists (SP and YN; both over 20 years of experience as abdominal radiologist). Observers were blinded to the preparation protocol, date of examination, and clinical information, with the exception of surgical history. In addition, scans were anonymized and randomized.

**Fig. 1:**
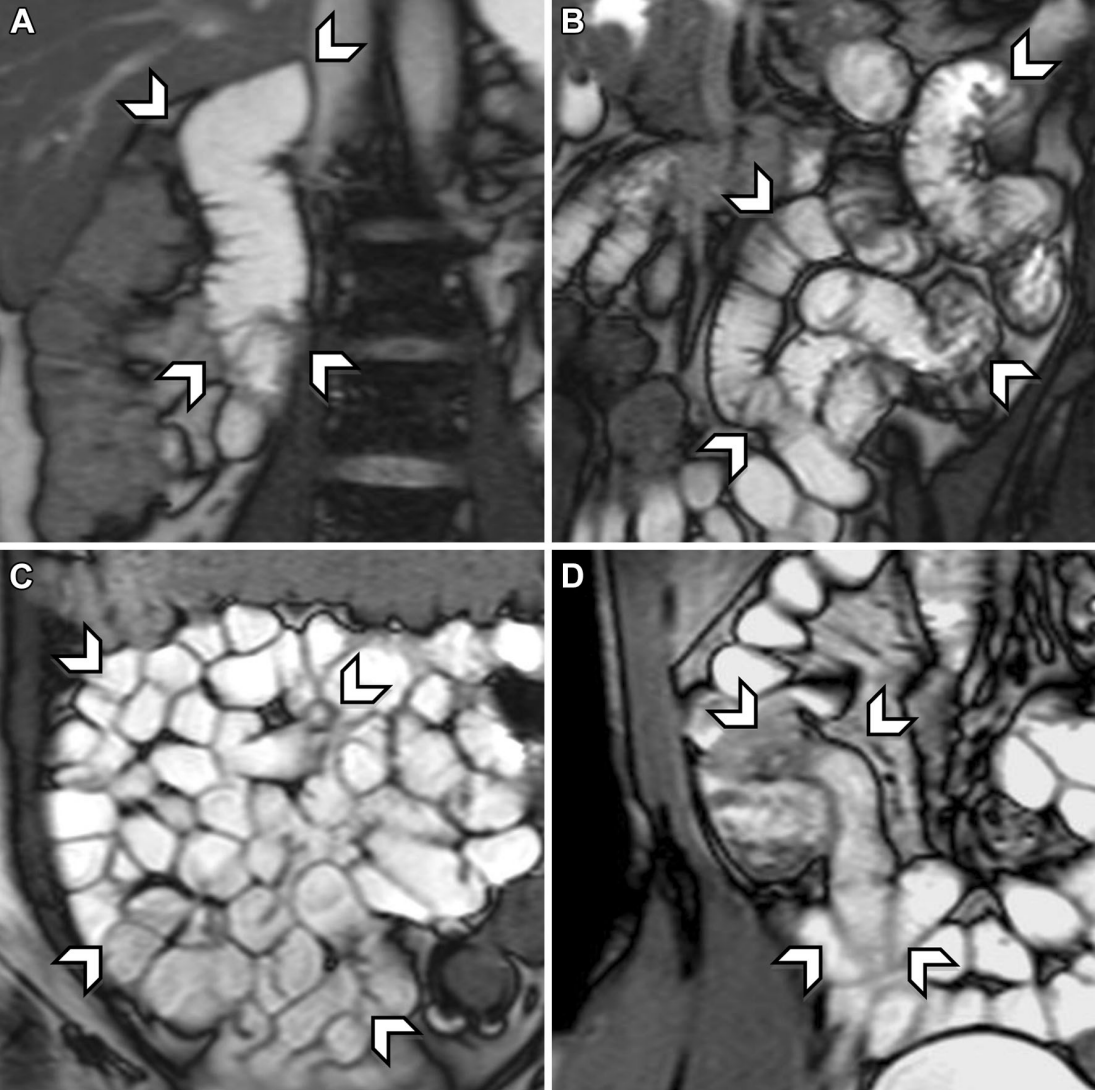
Coronal-balanced gradient-echo sequences with fat saturation show examples of optimal distension of the four small bowel segments in different patients. From *top left* to *bottom right* all figures show examples of optimal distension, grade score 3 (>75% of segment adequately distended). **A** duodenum, **B** jejunum and **C** ileum, and **D** terminal ileum

**Fig. 2:**
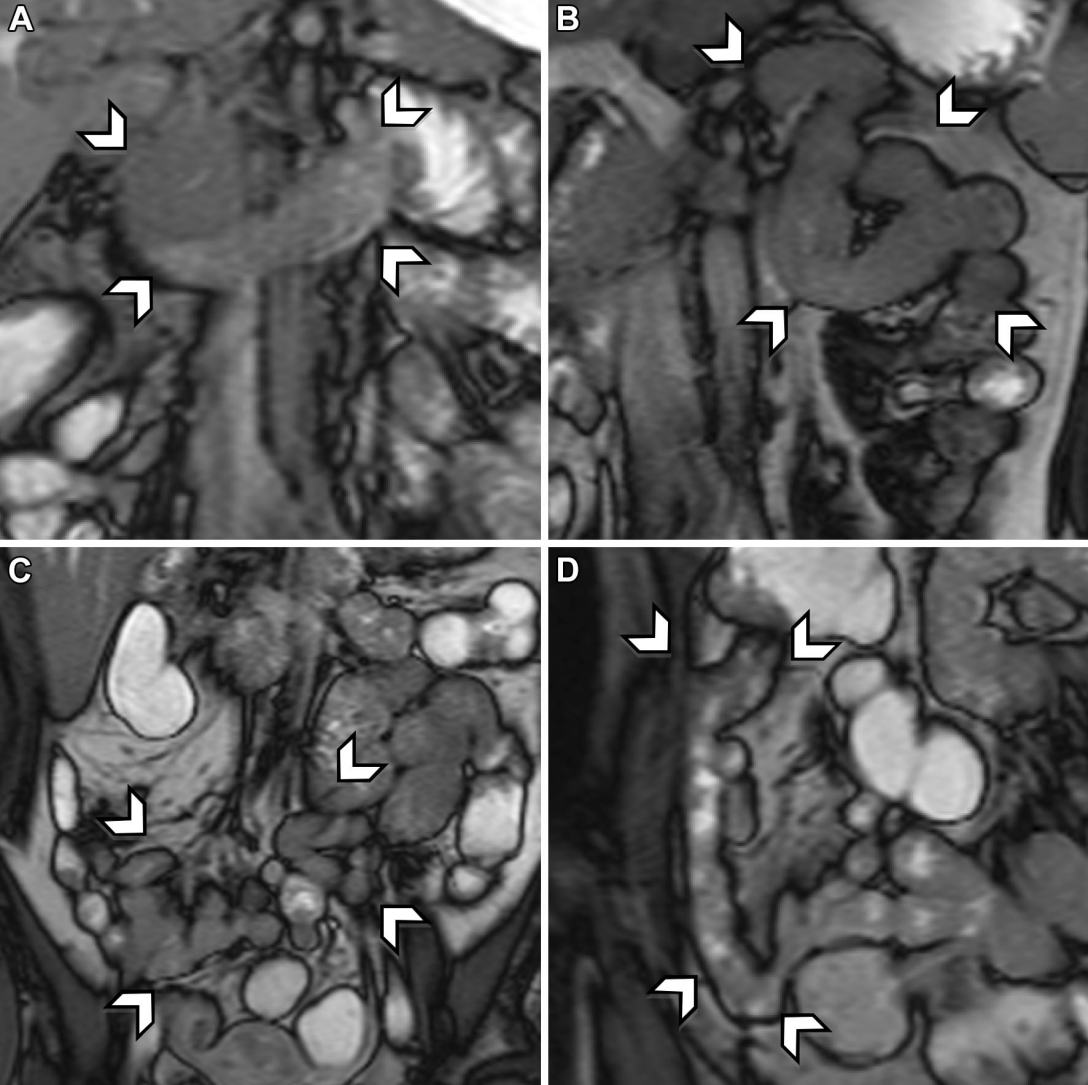
Coronal-balanced gradient-echo sequences with fat saturation show examples of inadequate distension of the four small bowel segments in different patients. Inadequate distension is score 0 for no distension or collapsed segment (<25% of segment adequately distended) or score 1 for insufficient distension (25%–50% of segment adequately distended). From *top left* to *bottom right* **A** duodenum grade 1, **B** jejunum grade 0, **C** ileum grade 1, and **D** neo-terminal ileum grade 1

Firstly, the stomach filling was evaluated. This was done to evaluate the pre-small bowel amount of luminal contrast medium. Stomach filling was scored for the gastric antrum as compared to the corpus on a three-grade score: (0) empty stomach, (1) normal filling, and (2) full stomach. Secondly, the small bowel was divided in four segments: the duodenum (identified by the “C” shape up to Treitz ligament), jejunum (left upper diagonal half of the abdomen), ileum (right lower diagonal half of the abdomen), and (neo-) terminal ileum (most distal 20 cm of the ileum). Each segment was evaluated using a four-grade score: (0) no distension or collapsed segment (<25% of segment adequately distended), (1) for insufficient distension (25%–50% of segment adequately distended), (2) for sub-optimal distension (50%–75% of segment adequately distended), and (3) optimal distension (>75% of segment adequately distended). See Figs. 1 and 2 for examples of different distension grades of the four small bowel segments. Lastly, the overall quality of the scan was assessed using a four-grade score (0–3): (0) for non-diagnostic quality, (1) diagnostic quality with numerous artifacts, (2) diagnostic with a few artifacts, and (3) diagnostic with no artifacts. All evaluations were performed using the medical image viewer Horos, version 1.1.7 (www.horosproject.org, GNU Lesser General Public License, Version 3.0).

### Statistical analysis

To obtain the best estimate for bowel segment distension, values were averaged between both observers for each segment and total small bowel distension. Total small bowel distension was calculated as the sum of all small bowel segment scores (duodenum, jejunum, ileum, and (neo-) terminal ileum). For both patient groups, mean distension scores with corresponding confidence intervals were calculated separately for both observers and for the averaged values of both observers for each segment and total small bowel. Differences in distension scores and disease activity between groups were analyzed using the *χ* ^2^ test for ordinal data. A *p* value less than 0.05 was considered to indicate a statistically significant difference. The degree of interobserver agreement for the separate segments was assessed using Cohen’s kappa with linear weighting and for total small bowel distension using the intraclass correlation coefficient (ICC). Kappa values and ICC’s were interpreted as follows: poor (<0.00), slight (0.00–0.20), fair (0.21–0.40), moderate (0.41–0.60), substantial (0.61–0.80), and almost perfect (0.81–1.00) [13]. Statistical analysis was performed using IBM SPSS Statistics Version 23 (IBDM corporation, Armonk, NY, USA).

## Results

### Patients

A total of 105 patients were found eligible for inclusion, of which five patients were excluded for the following reasons: patients with multiple scans during the inclusion period (*n* = 2) and patients who could not comply with the oral preparation protocol (*n* = 3). One patient started vomiting within 10 min after start of oral contrast ingestion and this patient was further unable to retain oral contrast. Additionally, two patients received oral contrast more than 2 h before the start of the examination due to a scheduling error. To compensate for the extended time, these patients received an extra amount of oral contrast outside of our study protocol. Finally, 50 patients were included in the 60-min preparation group (28 female, median age 40 years, interquartile range (IQR) 23–51) and 50 patients in the 45-min preparation group (29 female, median age 37 years, IQR 30–54). Patient characteristics for both groups are given in Table 2.

**Table 2:** Patient characteristics

	60-min preparation	45-min preparation
Patients, *n*	50	50
Female, *n* (%)	28 (56%)	29 (58%)
Age at time of MRI examination in years, median (IQR)	40 (23–51)	37 (30–54)
Gastrointestinal surgical procedure, *n* (%)	22 (44%)	27 (54%)
Small bowel disease*		
No disease activity, *n* (%)	13 (26%)	22 (44%)
Disease activity without stenosis, *n* (%)	27 (54%)	13 (26%)
Disease activity with stenosis, *n* (%)	10 (20%)	15 (30%)
Drinking time in minutes, median (IQR)	**	45 (44–50)***
Volumes of oral contrast administered		
Portion one (0–20 min) in mL, median (IQR)	****	700 (588–700)
Portion two (20–40 min) in mL, median (IQR)	****	563 (450–700)
Portion three (directly before scan) in mL, median (IQR)	****	150 (100–200)
Total volume in mL, median (IQR)	****	1400 (1192–1600)

n, number; IQR, interquartile range; mL, milliliter; *, Data from clinical radiological report; **, The exact drinking times were not documented as these retrospective group examinations had been performed prior to initiation of this study. However, by subtraction of the administration time at the MRI-unit and the time at the start of the examination, an estimate can be made that ten patients had a drinking time of less than 60 min. The maximum drinking times from these ten patients from high to low were 35, 44, 48, 53, 54, 54, 56, 57, 58, and 59 min. From the other 40 patients we can determine that they arrived at the MRI-unit at least 60 min before the examination; ***, Five patients had an administration time more than 60 min due to unforeseeable circumstances at MRI-unit. The administration times of these patients were 61, 64, 65, 73, and 73 min; ****, Data not documented as these retrospective group examinations had been performed prior to initiation of this study

In the 60-min group, 22 patients had undergone previous bowel resection surgery, while 37 patients showed small bowel disease activity, of which 10 had stricturing disease. In the 45-min group, 27 patients had undergone previous bowel resection surgery, while 28 patients showed small bowel disease activity, of which 15 patients had stricturing disease. No significant differences in disease activity were seen between both groups (*p* = 0.6).

Exact drinking times and administered volume were not documented for the 60-min group as these examinations had been performed prior to initiation of this study. However, an adequate estimation of drinking time could be made. Using the arrival registration times and time of the start of the scan, it was evident that ten patients in the 60-min preparation group had drinking times under 60 min (range 35–59), of whom three had less than 50-min drinking time (35, 44 and 48 min). In the prospective 45-min group, the median drinking time was 45 min (IQR 44–50 min). Five patients in that group had a preparation time longer than 60 min (61, 64, 65, 73, and 73 min). From portion one, two, and three, patients consumed a median volume of 700 mL (IQR 588–700 mL), 563 mL (IQR 450–700 mL), and 150 mL (IQR 100–200 mL), respectively. The median total ingested volume was 1400 mL (IQR 1192–1600 mL).

### Segmental distension

A total of 500 segments (100 stomach, 400 small bowel) were scored by two observers. All examinations were described as diagnostic by both observers, showing mean quality scores of 2.8 and 2.2 for the retrospective group and 2.8 and 2.1 for the prospective group, respectively. Results for segment distension are presented in Table 3. The following segments distension scores showed a significant difference by one of both observers in favor of the 45-min protocol: the stomach (*p* = 0.04), duodenum (*p* = 0.02), jejunum (*p* = 0.02), and total small bowel (*p* = 0.02). In the averaged values, significant differences in favor of the 45-min protocol were seen for the stomach (*p* = 0.04), jejunum (*p* = 0.02), and total small bowel (*p* = 0.02). No significant differences were found in favor of the 60-min preparation. This analysis was repeated with exclusion of eight patients with strongly divergent preparation times (see patients paragraph). The repeated analysis showed no differences considering significant differences, although already significant differences showed further decrease in *p* value. Furthermore, all except the mean distension score of the duodenum by one observer was higher in the 45-min preparation, compared to the 60-min preparation. The interobserver agreement from stomach, duodenum, jejunum, ileum, (neo-) terminal ileum, and total small bowel, respectively, were moderate (*κ* = 0.47), slight (*k* = 0.19), fair (*k* = 0.31), slight (*k* = 0.17), fair (*k* = 0.34), and moderate (ICC = 0.40). The following number of discrepant scores was found for each segment (>1 grade discrepancies/total discrepancies): stomach (0/34), duodenum (5/47), jejunum (3/43), ileum (11/71), and (neo-) terminal ileum (10/49).

**Table 3:** Distension ratings per-segment and total small bowel, interobserver agreement, and average score by the observers

Segment	Observer one		*p* value*	Observer two		*p* value*	Interobserver agreement	Average score observers		*p* value*
	60-min preparation mean (95% CI)	45-min preparation mean (95% CI)		60-min preparation mean (95% CI)	45-min preparation mean (95% CI)		Weighted kappa mean (95% CI)	60-min preparation mean (95% CI)	45-min preparation mean (95% CI)	
Stomach	**1.50 (1.36**–**1.64)**	**1.70 (1.57**–**1.83)**	**0.04**	1.18 (0.98–1.38)	1.42 (1.24–1.60)	0.08	0.47 (0.36–0.59)	**1.34 (1.18**–**1.50)**	**1.56 (1.42**–**1.70)**	**0.04**
Duodenum	1.60 (1.41–1.79)	1.58 (1.38–1.78)	0.88	**1.16 (1.02–1.30)**	**1.40 (1.25–1.55)**	**0.02**	0.19 (0.06–0.32)	1.38 (1.25–1.51)	1.49 (1.35–1.63)	0.25
Jejunum	1.46 (1.26–1.66)	1.70 (1.52–1.88)	0.08	**1.24 (1.09–1.39)**	**1.54 (1.32**–**1.76)**	**0.02**	0.31 (0.15–0.46)	**1.35 (1.21**–**1.49)**	**1.62 (1.45**–**1.79)**	**0.02**
Ileum	2.48 (2.30–2.66)	2.52 (2.35–2.69)	0.75	1.64 (1.44–1.84)	1.80 (1.59–2.01)	0.26	0.17 (0.09–0.25)	2.06 (1.90–2.22)	2.16 (1.99–2.33)	0.39
(Neo-) terminal ileum	2.28 (1.99–2.57)	2.58 (2.37–2.79)	0.09	2.02 (1.78–2.26)	2.26 (2.03–2.49)	0.15	0.34 (0.20–0.48)	2.15 (1.92–2.38)	2.42 (2.24–2.60)	0.07
Total small bowel**	1.96 (1.82–2.09)	2.10 (1.97–2.22)	0.12	**1.52 (1.40**–**1.63)**	**1.75 (1.60**–**1.90)**	**0.02**	0.40 (0.02–0.64)***	**1.74 (1.62**–**1.85)**	**1.92 (1.81**–**2.04)**	**0.02**

Significant statistical differences in bold; * *χ* ^2^ test; ** total small bowel includes duodenum, jejunum, ileum and (neo-) terminal ileum; *** calculated with the intraclass correlation coefficient.

## Discussion

In this study, we found an improved overall small bowel distension and jejunal distension using the 45-min oral preparation protocol. Distension of the duodenum, ileum, and (neo-) terminal ileum showed no difference in favor of either preparation protocol. The interobserver agreement found in this study for the small bowel segments ranged from slight to moderate, although most discrepancies were small with one-grade differences, especially in the stomach, duodenum, and jejunum.

Several studies have evaluated the time needed to attain adequate and continuous distension for MR enterography in small groups of healthy participants [3, 12, 14]. To our knowledge, our study is the first to investigate preparation time needed in a large group of suspected and known Crohn’s disease patients, the main target group for MR enterography. Several studies on oral contrast preparation protocols for MR enterography have found proximal small bowel distension to be lower compared to distal small bowel distension [6, 12, 15]. Our findings confirm this for both preparation protocols as duodenum and jejunum distension were lower compared to distension of the ileum and (neo-) terminal ileum. Although an improvement in jejunal distension was found using the 45-min preparation, the resulting distension remained lower than that of the ileum and (neo-) terminal ileum. Although we assume clinical benefit from improved distension in the jejunum, the amount needed for clinically relevant improvement is unknown.

There are multiple options to grade small bowel distension. Although some studies have performed diameter measurements, we chose a qualitative grading approach to evaluate small bowel distension [3, 6]. Distension is often inconsistent over the length of a segment, for which localized diameter measurements will not provide a representative estimate. Multiple measurements could be used to obtain an overall estimate, although this is complicated by extensive surgical resections and resulting anatomic variations, which are common in Crohn’s disease patients. However, qualitative assessment is also prone to interobserver variability, as we have found in the current study. Differences in grading distension can arise from estimation of distension in long segments with varying distensions or because of varying interpretations of grading criteria. Taking this into consideration, we used averaged values of the observers to obtain the best estimate of bowel distension. In our opinion, this approach is justified in cases where evaluation of the measured outcome is the primary goal, instead of the grading system that was used. Furthermore, we chose not to exclude patients that were outliers for drinking times, as this reflects daily clinical practice.

Our study has several limitations. Firstly, part of our study population was retrospectively included. As a result, some data were not documented at the time, such as drinking times and ingested volume of oral contrast medium. Ideally, the latter should have been similar between both groups. Importantly, both included groups were comparable for sex, age, surgical history, and disease activity. Secondly, no use was made of a gastroprokinetic agent; this is also not a standard procedure in many other centers. A gastroprokinetic agent such as metoclopramide (intravenous) could be used to stimulate gastric emptying. As gastric filling was significantly higher for the 45-min group with the three-portion oral preparation, a gastroprokinetic could have potentially provided further improvement of proximal small bowel distension [16, 17]. Finally, we assumed that improved proximal small bowel distension will enhance diagnostic accuracy, as this is also seen in distal small bowel segments [18]. However, a study comparing MR enterography to MR enteroclysis described similar diagnostic accuracy in the terminal ileum, regardless of differences in distension [5]. In the same reasoning, improved proximal small bowel distension by an adapted oral protocol might not necessary lead to increased diagnostic accuracy. However, that earlier study has several limitations that might affect the validity of the results. Namely, none of the 40 included patients were found to have jejunal disease and thus, conclusions regarding diagnostic accuracy in the jejunum are limited. Furthermore, no suitable reference standard, such as double balloon enteroscopy or video capsule endoscopy was used, although both techniques also have limitations [19]. For these reasons, independent evaluation of bowel distension might be the preferable outcome measure compared to diagnostic accuracy in the proximal small bowel.

In conclusion, we advise to use a 45-min three-portion oral preparation for MR enterography with the benefit of improved overall small bowel distension, with an increase in proximal small bowel distension, and especially jejunal distension. Additionally, it is evident that a better time-efficiency is achieved using a 15-min shorter preparation protocol.

## Abbreviations

GE: Gradient echo
ICC: Intraclass correlation coefficient
IQR: Interquartile range
MR: Magnetic resonance
MRI: Magnetic resonance imaging
SPGE: Spoiled gradient echo
SSFSE: Single-shot fast spin echo
T1-w: T1 weighted MRI sequence
T2-w: T2 weighted MRI sequence
